# Variations in clinical decision-making between cardiologists and cardiac surgeons; a case for management by multidisciplinary teams?

**DOI:** 10.1186/1749-8090-1-2

**Published:** 2006-03-03

**Authors:** MA Denvir, JP Pell, AJ Lee, J Rysdale, RJ Prescott, H Eteiba, A Walker, P Mankad, IR Starkey

**Affiliations:** 1 Department of cardiology, Western General Hospital, Crewe Road, Edinburgh, EH4 2XU, UK; 2 Department of medical cardiology, University of Glasgow, 10 Alexandra Parade, Glasgow, G31 2ER, UK; 3 Medical Statistics Unit, University of Edinburgh, Teviot Place, Edinburgh, EH8 9AG, UK; 4 Department of statistics and health economics, University of Glasgow, University Avenue, Glasgow G12 8QQ, UK; 5 Department of Cardiac Surgery, Royal Infirmary of Edinburgh, Little France, UK

## Abstract

**Objective:**

To assess variations in decisions to revascularise patients with coronary heart disease between general cardiologists, interventional cardiologists and cardiac surgeons

**Design:**

Six cases of coronary heart disease were presented at an open meeting in a standard format including clinical details which might influence the decision to revascularise. Clinicians (n = 53) were then asked to vote using an anonymous electronic system for one of 5 treatment options: medical, surgical (CABG), percutaneous coronary intervention (PCI) or initially medical proceeding to revascularisation if symptoms dictated. Each case was then discussed in an open forum following which clinicians were asked to revote. Differences in treatment preference were compared by chi squared test and agreement between groups and between voting rounds compared using Kappa.

**Results:**

Surgeons were more likely to choose surgery as a form of treatment (p = 0.034) while interventional cardiologists were more likely to choose PCI (p = 0.056). There were no significant differences between non-interventional and interventional cardiologists (p = 0.13) in their choice of treatment. There was poor agreement between all clinicians in the first round of voting (Kappa 0.26) but this improved to a moderate level of agreement after open discussion for the second vote (Kappa 0.44). The level of agreement among surgeons (0.15) was less than that for cardiologists (0.34) in Round 1, but was similar in Round 2 (0.45 and 0.45 respectively)

**Conclusion:**

In this case series, there was poor agreement between cardiac clinical specialists in the choice of treatment offered to patients. Open discussion appeared to improve agreement. These results would support the need for decisions to revascularise to be made by a multidisciplinary panel.

## Background

Coronary revascularisation rates vary widely and are not explained by geographical variations in the incidence of coronary artery disease (CAD) [1-3]. Previous studies from North America have suggested that variations tend to reflect inappropriately high thresholds in low frequency areas, rather than overuse elsewhere [4]. A recent study suggested that under use of revascularisation results in preventable morbidity and mortality [5].

Following detection of CAD at coronary angiography, two decisions must be made: whether to attempt revascularisation and whether to do this via coronary artery bypass grafting (CABG) or percutaneous coronary intervention (PCI). Both decisions are commonly made by the cardiologist. Some have argued that this represents a conflict of interest for an interventional cardiologist [6,7] who acts as both "poacher" and "gamekeeper". This issue has become increasingly important as the use of PCI has grown and CABG rates have fallen [8].

We used standardised case scenarios to examine variations in decisions related to coronary revascularisation among cardiologists and cardiac surgeons.

## Methods

Six patients with coronary artery disease were presented in a standard format to 53 cardiac specialists at an open meeting. Cases were selected for presentation by a panel of cardiologists on the basis that they represented typical cases of coronary artery disease. Before any discussion or voting, cases were presented to the audience in a standard format including the clinical presentation, details of co-morbidity, medication and results of stress testing. Digital recordings of left ventriculography and coronary angiography were also presented (table 1). An electronic keypad voting system was used to identify the type of clinician present (non-interventional cardiologist, interventional cardiologist, cardiac surgeon, table 2) After each case presentation the individual clinician was asked to vote using the keypad for one of five treatment categories:

**Table 1:** Clinical details of the six cases as presented

**Patient**	**Age**	**Presentation**	**Stress Test**	**LV function**	**Severity CAD**	**Co-morbitity**	**Drug therapy**	**NZ score**	**ACRE score**
A	54	Unstable	Positive	Good	3VD	Mild asthma	Oral ×3	69	CABG
B	65	Stable	Positive	Good	3VD incl LMS	Obese	Oral ×2	60	CABG
C	74	Unstable	Not done	Good	3VD excl prox LAD	Rheumatoid	Oralx3	60	CABG or PCI
D	61	Stable	Positive	Good	3VD	Osteoarthritis	Oral ×2	65	CABG
E	64	Stable	Positive	Good	3VD	Obese, HBP	Oral ×2	30	CABG
F	67	Stable	Positive	Moderate	2VD excl prox LAD	None	Oral ×1	43	Uncertain

**Key **: Stress test – exercise test, positive is defined as >2 mm ST depression, CAD – coronary artery disease, VD – vessel disease, LMS – left main stem, LAD – left anterior descending artery, exc – excluding, prox – proximal, NZ score – New Zealand score (>35 merits revascularisation) [8], HBP – hypertension, Drug therapy – oral – 1,2 or 3 oral anti-anginal drugs, ACRE score (see reference 9), CABG – coronary artery by-pass grafting, PCI – percutaneous coronary intervention.

**Table 2:** Treatment preference by specialty in round 1 (numbers of individuals/cases)

	**Cardiac surgeons**	**Interventional cardiologists**	**Non-interventional cardiologists**	**Total**
CABG	25 (53%)	31 (40%)	56 (42%)	112 (43%)
PCI	5 (11%)	23 (30%)	28 (21%)	56 (22%)
Medical	17 (36%)	23 (30%)	50 (37%)	90 (35%)
Missing	1	7	52	60
Total	48	84	186	318

1. coronary artery by-pass grafting, 2. medical treatment initially with a view to CABG if symptoms dictated, 3. coronary angioplasty, 4. medical treatment initially with a view to PCI if symptoms dictated, 5. medical therapy. Cases were presented in succession with voting after each presentation until all 6 had been presented, initially with no discussion. Each case was then discussed in an open forum led by a panel of experts with the audience asked to participate in the discussion. The cases were then presented for a second time and everyone was asked to revote on their treatment category (1–5) for each case. The responses by the participants have also been compared to treatment recommendations based on the New Zealand score [9] (score greater than 35 points merits revascularisation) and the ACRE study which used a multidisciplinary panel to rate the criteria for selecting patients for coronary revascularisation [10].

### Statistical analysis

The responses for each individual were summed over each response category, so that each individual (who answered all six Round 1 questions) contributes 6 observations to the analysis. Due to the small number of participants in certain response categories, some analysis used grouped responses. The "medical" treatments were grouped together and compared with angioplasty and surgery separately, and angioplasty and surgery together. The occupations "cardiologist" and "other cardiologists/physicians with cardiology interest" were grouped into "non-interventional cardiologists". Results were compared by the χ^2 ^test. Round 1 responses were compared to Round 2 for each of the six cases. The weighted Kappa statistic was employed for analysis as it has been specifically designed to measure the level of agreement between two raters or evaluators. However, it was also used to measure the level of agreement between Round 1 and Round 2. A Kappa statistic of 0.00–0.40 suggests "poor" agreement, 0.41–0.60 "moderate" agreement, 0.61–0.75 "substantial" agreement, 0.76–0.99 "excellent" agreement and 1.00 represents "perfect" agreement [11].

## Results

Surgeons were more likely to choose surgery as a form of treatment (table 2, P = 0.034,) while interventional cardiologists were more likely to choose PCI (P = 0.056). There were no differences in the form of treatment selected by non-interventional cardiologists compared with interventional cardiologists (P = 0.13).

There was poor agreement between all clinicians in the first round of voting (table 3, Kappa 0.26) but this improved to a moderate level of agreement after open discussion for the second vote (Kappa 0.44). The level of agreement among surgeons (0.15) was less than that for cardiologists (0.34) in Round 1, but was similar in Round 2 (0.45 and 0.45 respectively).

**Table 3:** Mean preferred category of treatment (score of 1 = definite surgery and 5 = definite medical therapy, see methods section) and agreement of voting between rounds for all specialists (weighted Kappa,) for each of the six case scenarios

	**Mean preferred treatment category**		**Kappa (round 1 vs round 2)**
	**Round 1**	**Round 2**	
Patient A	2.8	3.3	0.47
Patient B	1.3	1.2	0.22
Patient C	2.4	2.9	0.24
Patient D	1.2	1.3	0.15
Patient E	3.5	3.8	0.59
Patient F	2.7	1.7	0.51

Two patients (C and E) produced significant differences in treatment preference between round 1 and round 2 of voting suggesting that the discussion between rounds had produced an overall change in clinical opinion (table 3). Both of these patients had significant co-morbidity which may have influenced the choice of treatment with one swinging from predominantly surgery to angioplasty and the other from an equal number of angioplasty and medical to predominantly medical therapy. Overall, comparing the pattern of votes between rounds 1 and 2, there was a small non-significant reduction in the number of votes for medical therapy alone with an increased number of votes for medical therapy followed by either PCI or CABG if symptoms dictated and no significant increase in the overall use of revascularisation (table 4).

**Table 4:** Comparison of treatment category between voting in round 1 and round 2 (N = number of votes for all 6 case scenarios)

**Treatment choice**	**Round 1 N**	**Round 2 N**	**Total N**
Surgery	151	141	292
Medical/surgery	27	23	50
Angioplasty	66	70	136
Medical/angioplasty	49	68	117
Medical only	24	13	37
Total	317	315	632

χ^2 ^test: 7.24, p = 0.124

There was no relationship observed between the New Zealand score and the likelihood of agreement between clinicians. Despite a New Zealand score for all six cases which merited revascularisation there were a significant number of decisions to manage patients medically in more than one third of cases (35%). This was consistent between cardiologists (35%) and cardiac surgeons (36%).

## Discussion

There is no widely accepted clinical pathway for assessing CHD patients for revascularisation following coronary angiography. In tertiary centres with cardiac surgery on-site, the cardiologist may have the opportunity to discuss the case and angiographic findings directly with a cardiac surgeon. This can occur on an ad hoc or a formal basis such as at an open clinical meeting. Rarely does this occur in a dynamic, daily, case by case basis. For cardiologists working in non-surgical centres discussion with a cardiac surgeon is often more difficult and dynamic discussion may not take place. In all situations the cardiologist commonly acts as the gatekeeper for angiography and revascularisation. New developments in information technology with high speed electronic digital links may facilitate formal interactive discussion between specialists but such systems are not yet widely available. Such discussions should not take place with the patient lying waiting on the cardiac catheter laboratory table.

This study highlights a number of issues. Firstly, some variation in the use of coronary revascularisation therapy is due to variations between individual clinician's working practices. Secondly, this variation was greatest between surgeons and interventional cardiologists. This supports findings in other areas of clinical medicine where clinical specialists who perform a procedure are more likely to consider it to be appropriate in certain case scenarios [12].

Cardiologists have, at best, only moderate agreement among themselves in the form of treatment chosen for these six cases despite all six scoring highly using the New Zealand system and using the criteria from the ACRE study. Cardiologists clearly add variability to the decision process even before discussion with a surgeon has taken place. Surgeons had even less agreement among themselves at baseline. The combined effect of these variations could account for a significant degree of variability in use and choice of revascularisation therapy in routine clinical practice.

Discussion between rounds appeared to improve agreement among surgeons and cardiologists although the choice of treatments did not change significantly. This indicates that such discussion may, at least, reduce the variability of decision-making but may not change the overall utilisation of revascularisation therapy. Similar discussions might produce more consistent decisions in clinical practice but may be practically difficult within current systems of healthcare. Ideally the main carers for a patient such as the community practitioner, the cardiologist and a cardiac surgeon would represent the professional core of the discussion group with the patient and, ideally, a close family member also being involved. This might be particularly useful in clinical scenarios where indications for revascularisation were strong but where there was significant co-morbidity which influenced the decision-making process. While this could be done for elective cases it would be very difficult to do in emergency situations.

Similar multidisciplinary approaches to clinical decisions are now widely used in oncology [13-15] and have been proposed previously for cardiac revascularisation [16,17]. As interventional cardiologists increasingly treat patients with multi-vessel disease [18] the need to carefully assess the range of options available to the patient is extremely important.

## Conclusion

In this typical case series, there was poor agreement between cardiac clinical specialists in the choice of treatment offered to patients. Open discussion appeared to improve agreement. These findings strongly support the need for decisions to revascularise patients to be made by a multidisciplinary panel using agreed criteria.
